# Newborn Cry Acoustics in the Assessment of Neonatal Opioid Withdrawal Syndrome Using Machine Learning

**DOI:** 10.1001/jamanetworkopen.2022.38783

**Published:** 2022-10-27

**Authors:** Andrew W. Manigault, Stephen J. Sheinkopf, Harvey F. Silverman, Barry M. Lester

**Affiliations:** 1 Brown Center for the Study of Children at Risk, Women & Infants Hospital of Rhode Island, Providence; 2 Thompson Center for Autism and Neurodevelopmental Disorders, University of Missouri, Columbia; 3 School of Engineering, Brown University, Providence, Rhode Island; 4 Department of Psychiatry, Alpert Medical School of Brown University, Providence, Rhode Island; 5 Department of Pediatrics, Alpert Medical School of Brown University, Providence, Rhode Island

## Abstract

**Importance:**

The assessment of opioid withdrawal in the neonate, or neonatal opioid withdrawal syndrome (NOWS), is problematic because current assessment methods are based on subjective observer ratings. Crying is a distinctive component of NOWS assessment tools and can be measured objectively using acoustic analysis.

**Objective:**

To evaluate the feasibility of using newborn cry acoustics (acoustics referring to the physical properties of sound) as an objective biobehavioral marker of NOWS.

**Design, Setting, and Participants:**

This prospective controlled cohort study assessed whether acoustic analysis of neonate cries could predict which infants would receive pharmacological treatment for NOWS. A total of 177 full-term neonates exposed and not exposed to opioids were recruited from Women & Infants Hospital of Rhode Island between August 8, 2016, and March 18, 2020. Cry recordings were processed for 118 neonates, and 65 neonates were included in the final analyses. Neonates exposed to opioids were monitored for signs of NOWS using the Finnegan Neonatal Abstinence Scoring Tool administered every 3 hours as part of a 5-day observation period during which audio was recorded continuously to capture crying. Crying of healthy neonates was recorded before hospital discharge during routine handling (eg, diaper changes).

**Exposures:**

The primary exposure was prenatal opioid exposure as determined by maternal receipt of medication-assisted treatment with methadone or buprenorphine.

**Main Outcomes and Measures:**

Neonates were stratified by prenatal opioid exposure and receipt of pharmacological treatment for NOWS before discharge from the hospital. In total, 775 hours of audio were collected and trimmed into 2.5 hours of usable cries, then acoustically analyzed (using 2 separate acoustic analyzers). Cross-validated supervised machine learning methods (combining the Boruta algorithm and a random forest classifier) were used to identify relevant acoustic parameters and predict pharmacological treatment for NOWS.

**Results:**

Final analyses included 65 neonates (mean [SD] gestational age at birth, 36.6 [1.1] weeks; 36 [55.4%] female; 50 [76.9%] White) with usable cry recordings. Of those, 19 neonates received pharmacological treatment for NOWS, 7 neonates were exposed to opioids but did not receive pharmacological treatment for NOWS, and 39 healthy neonates were not exposed to opioids. The mean of the predictions of random forest classifiers predicted receipt of pharmacological treatment for NOWS with high diagnostic accuracy (area under the curve, 0.90 [95% CI, 0.83-0.98]; accuracy, 0.85 [95% CI, 0.74-0.92]; sensitivity, 0.89 [95% CI, 0.67-0.99]; specificity, 0.83 [95% CI, 0.69-0.92]).

**Conclusions and Relevance:**

In this study, newborn acoustic cry analysis had potential as an objective measure of opioid withdrawal. These findings suggest that acoustic cry analysis using machine learning could improve the assessment, diagnosis, and management of NOWS and facilitate standardized care for these infants.

## Key Points

**Question:**

Can newborn cry acoustics serve as an objective biobehavioral marker of neonatal opioid withdrawal syndrome (NOWS)?

**Findings:**

In this cohort study of 65 neonates with and without exposure to opioids, supervised machine learning methods identified a set of cry acoustic parameters that accurately predicted which infants received pharmacological treatment for NOWS, with an area under the curve of 0.90, accuracy of 0.85, sensitivity of 0.89, and specificity of 0.83.

**Meaning:**

These results suggest that acoustic cry analysis using machine learning has potential as a measure of opioid withdrawal in neonates.

## Teaser

This cohort study evaluates whether newborn acoustic cry analysis using machine learning is feasible as a measure of neonatal opioid withdrawal syndrome.

## Introduction

Opioid use during pregnancy has reached epidemic proportions, with a 242% increase in the past 10 years,^1^ resulting in increases in the prevalence of neonatal opioid withdrawal syndrome (NOWS), also known as neonatal abstinence syndrome.^2,3^ NOWS is a substantial public health problem, increasing from 1.6 per 1000 births in 2004 to 8.8 per 1000 births in 2016,^4^ with associated hospital charges increasing from $190 million in 2000 to $720 million in 2009. Clinical signs of NOWS reflect opioid receptor expression^5^ and include dysfunction of the central, autonomic, respiratory, and gastrointestinal systems (eg, tremors, excessive and/or high-pitched crying, and breathing and intestinal problems).^6^

Focused attention on NOWS has highlighted the assessment and management of this condition, with implications ranging from immediate use of pharmacological treatment to subsequent development^7,8^ and survival.^9^ Absent biological markers, the treatment of NOWS is based on observer-rated scales to evaluate NOWS severity and the need for pharmacological treatment. Until recently, the gold standard for NOWS assessment has been the Finnegan Neonatal Abstinence Scoring Tool (FNAST).^10^ The FNAST includes 21 items (score range, 0-46, with higher scores indicating more severe withdrawal symptoms) and was developed as a clinical aid to assess NOWS severity and guide pharmacological treatment. Although the FNAST, including various modifications, is used in 95% of institutions in the US,^11,12,13^ the literature is replete with frustration and dissatisfaction with the FNAST, including the length,^14,15^ subjectivity,^16^ validity, and reliability^17^ of the tool and the need to disturb infants to perform formal assessments.^18^ There are also concerns that the FNAST overestimates the need for pharmacological treatment by including signs that may not be clinically meaningful, resulting in increased length of hospital stay and hospital costs.^18^

The Eat, Sleep, Console (ESC) care tool^18,19^ was developed as an alternative to the FNAST, with the goal of reducing pharmacological treatment. The ESC focuses on nonpharmacological strategies as first-line treatment for infants with NOWS. If the infant meets criteria for sleeping, eating, and consoling, pharmacological treatment is not initiated or escalated. Early reports from researchers using the ESC tool have been favorable in terms of decreasing the use of pharmacological intervention and reducing length of stay; however, most studies have been retrospective, few psychometric data are available, and there has been no evidence that one approach is better than the other.^20^

Crying is a distinctive component in both the FNAST and ESC tools but is one of the most inadequately measured symptoms on the FNAST^21^ because the health care professional has to judge whether the cry is high pitched (not defined) and/or whether the infant is inconsolable. Inconsolability is also 1 of the 3 ESC criteria. A recent expert panel tasked with developing a standardized clinical definition of NOWS identified excessive crying as 1 of 5 clinical indicators used to support diagnosis.^22^ Crying may be important to the assessment of NOWS because infant cry characteristics reflect opioid receptor expression through the involvement of the brain stem (cranial nerves IX-XII), affecting the vocal tract, respiration, and gut.^23^ In addition, variation in cry acoustics (defined as the physical properties of sound) has been associated with the gene expression related to stress response systems.^24^ These pathways determine not only the regulatory behavior of cries, such as consolability, but also the acoustic characteristics of the cry (eg, pitch), many of which cannot be detected by human perception (ie, hearing) but can be measured objectively and reliably and reflect the pathophysiological features of withdrawal.

The purpose of the present cohort study was to evaluate the feasibility of using newborn cry acoustics as an objective biobehavioral marker of NOWS. We applied a machine learning approach to assess the accuracy with which acoustic cry characteristics predict the receipt of pharmacological treatment.

## Methods

### Study Design

This prospective controlled cohort study examined the association between acoustic cry characteristics and receipt of pharmacological treatment. The study was approved by the institutional review board of the Women & Infants Hospital of Rhode Island. Written informed consent was obtained; mothers provided consent on behalf of their infants after reviewing the consent form in the presence of trained research staff. Mothers consented prenatally, after delivery, or any time before the neonate would have met criteria for a diagnosis of neonatal abstinence syndrome; consent took place after hospital staff confirmed that mothers were capable of providing informed consent. This study followed the Strengthening the Reporting of Observational Studies in Epidemiology (STROBE) reporting guideline for cohort studies.

### Inclusion and Exclusion Criteria

A total of 177 full-term neonates were recruited at Women & Infants Hospital between August 8, 2016, and March 18, 2020 (eFigure in the Supplement). Data collection ended due to the COVID 19 quarantine. Inclusion criteria were (1) neonates older than 37 weeks’ gestation at birth, (2) English- or Spanish-speaking parents 18 years or older and able to give informed consent, (3) mothers receiving medication-assisted treatment (MAT) with methadone or buprenorphine, or (4) mothers not receiving MAT who did not use any illicit substances during pregnancy. Exclusion criteria were (1) mothers not receiving MAT who used any illicit substance during pregnancy and (2) neonates with sepsis, major congenital anomalies, or genetic disorders or neonates receiving care in the neonatal intensive care unit.

### Measures

Demographic and medical information was collected from electronic medical records. Neonates exposed to opioids were monitored for 5 days for signs of NOWS using the FNAST administered every 3 hours by trained nursing staff. Pharmacological treatment (morphine) was initiated when the FNAST score was 8 or higher on 2 consecutive assessments or 12 or higher on a single assessment. Neonates who did not meet criteria for pharmacological treatment by 5 days were discharged from the hospital. Healthy neonates were observed during routine handling (eg, diaper changes, bathing, or feeding).

Crying was recorded by attaching a digital audio recorder with an omnidirectional microphone to the side of the infant’s crib at a standardized location oriented toward the infant’s mouth. For neonates exposed to opioids, recording lasted from the beginning of the 5-day observation period until the infant was discharged; during this period, spontaneous and elicited cry episodes were recorded. For healthy neonates not exposed to opioids, crying was recorded during routine handling. Episodes of cry vocalizations were identified from the audio recordings; start time of the episode was used to determine whether pharmacological treatment had begun. Recordings were screened for sounds that could interfere with the acoustic analysis (ie, adult talking or environmental noises). Two research assistants, blinded to the prenatal drug history of the infant, were trained to identify cries appropriate for acoustic analysis and achieved 89% agreement.

A total of 177 neonates were recruited in the study, and cry recordings were processed for 118 neonates, 31 of whom produced cry recordings that were inappropriate for acoustic analysis (eFigure in the Supplement). Another 10 neonates were excluded from the study because they had no usable cries before treatment was initiated, and an additional 12 were removed because of missing data. The remaining 65 neonates included 19 infants with mothers receiving MAT who received pharmacological treatment for NOWS, 7 infants with mothers receiving MAT who did not develop NOWS, and 39 infants with no prenatal exposure to illicit substances.

**Figure 1.**
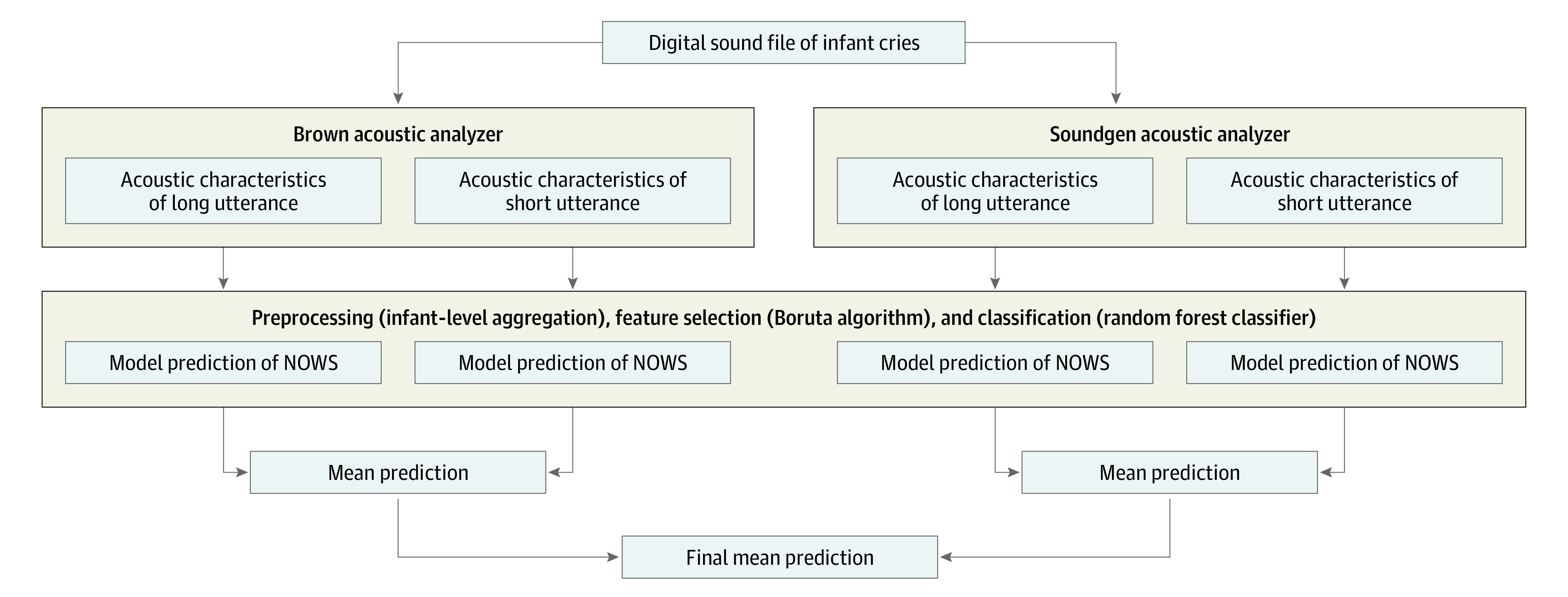
Model Stacking Diagram NOWS indicates neonatal opioid withdrawal syndrome.

Two acoustic analysis systems were used for the cry analysis: (1) a proprietary analysis software developed at Brown University (Brown analyzer) designed to perform cry analysis in infants^25^ and (2) an open-source analyzer developed for a broad range of applications, including analysis of human nonverbal vocalization (Soundgen^26^ package for R software, version 4 [R Foundation for Statistical Computing]). Using multiple analyzers via model stacking (ie, training models in parallel and combining their outputs) allows for a more comprehensive investigation of acoustic parameters, including the opportunity to identify common acoustic parameters across analyzers (Figure 1). Both analyzers were run in 2 phases. First, a cepstral-based acoustic analysis was used to extract acoustic parameters in 12.5-millisecond frames. Second, the acoustic parameters were organized and summarized into cry utterances. Cry utterances are cries that occur during the expiratory phase of respiration and include both short (<500 milliseconds) and long (≥500 milliseconds) utterances. The Brown analyzer produced 61 acoustic characteristics per utterance and identified 29 155 utterances (20 477 short and 8678 long); the Soundgen analyzer produced 55 acoustic characteristics per utterance and identified 14 061 utterances (6509 short and 7552 long). Short and long utterances were analyzed separately. For each newborn or acoustic measure, we computed means and rates of missing data. A total count of utterances was computed for each infant. A filtered subset of acoustic characteristics was generated by removing variables with high rates (>60%) of missing data, near zero variance,^27^ or excessive intercorrelation (*r* > .75),^27^ consistent with guidelines for machine learning feature filtering.^28^

### Statistical Analysis

**Figure 2.**
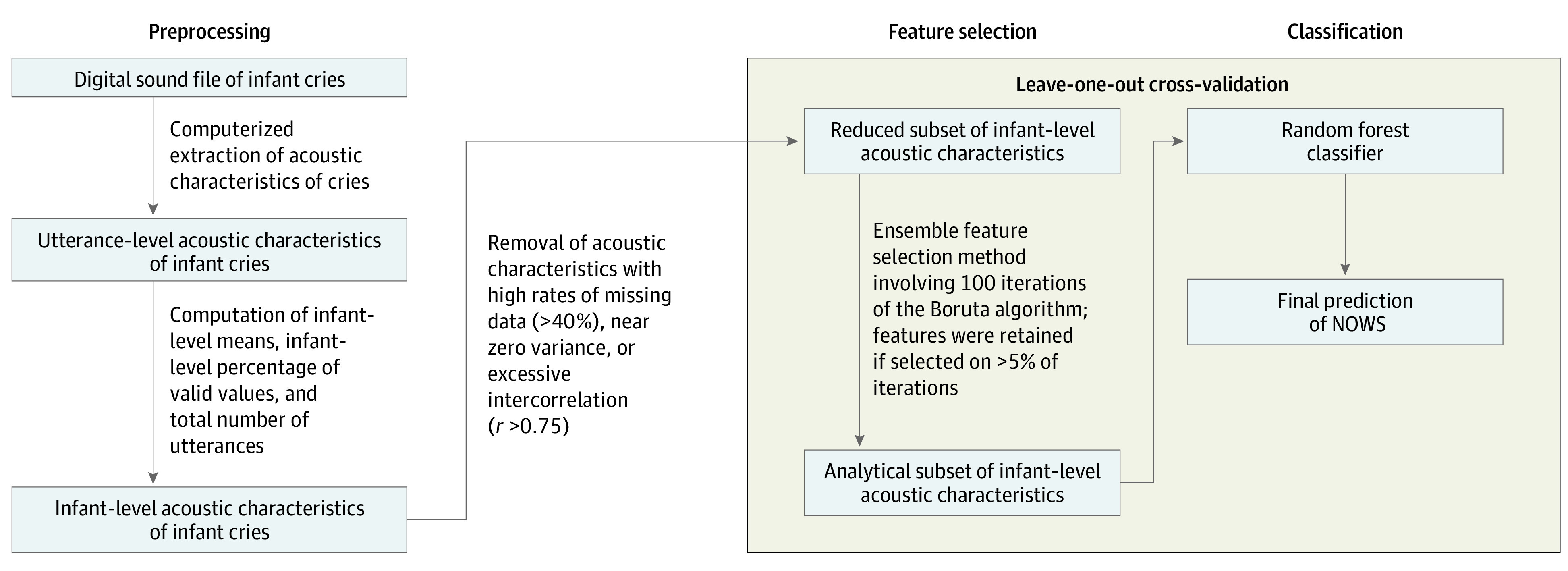
Flowchart of Data Reduction Strategy NOWS indicates neonatal opioid withdrawal syndrome.

We used cross-validated supervised machine learning methods in which random forest algorithms were trained to predict receipt of pharmacological treatment for NOWS using cry acoustics, and the accuracy of the algorithm was evaluated by generating a prediction on an unseen (or test) set. Leave-one-out cross-validation (LOOCV) was used to evaluate whether model predictions generalized to unseen cases. With this method, the test-train split (ie, the data split into a testing and training set) is repeated for every child in the data set such that the modeling procedure is performed for all except 1 child, and the held-out child is used to evaluate whether model predictions generalize to unseen cases. Both feature selection and classification steps were included in LOOCV to minimize bias in performance estimates^29^ (Figure 2).

An ensemble feature selection method^30^ was used to select acoustic cry variables where, within a single iteration of LOOCV, the Boruta algorithm^31^ was repeated 100 times, and variables selected by Boruta on greater than 5 of 100 times were retained for subsequent classification. Boruta is a performant^32^ algorithm that uses random forest to identify a subset of important predictors for a given outcome. Including feature selection within cross-validation allows for feature stability to be estimated, with 100% stability indicating that a given acoustic feature was always retained during feature selection. The relative contribution of each variable to the final prediction is measured with mean importance estimates. The mean of the predicted probabilities obtained from each model (Brown vs Soundgen and short vs long utterances) were used to generate a combined prediction of NOWS (Figure 1). Binary predictions were generated by finding the point on the receiver operating characteristic (ROC) curve exhibiting the shortest distance to perfect prediction.^33^ Standard diagnostic accuracy estimates were reported.^34^

Model predictions were examined in 3 ways. First, we computed the accuracy of models comparing neonates treated for NOWS with a control group comprising neonates with mothers receiving MAT who did not develop NOWS and healthy neonates who were not exposed to opioids. Next, we recomputed the accuracy estimates by comparing neonates who developed NOWS vs neonates with mothers receiving MAT who did not develop NOWS, then neonates who developed NOWS vs neonates with no prenatal illicit substance exposure. In this way, we were able to evaluate whether the results were biased toward either of the groups without NOWS.

In summary, we evaluated predictions of a single stacked model (ie, a meta-learner) consisting of 4 random forest classifiers (Figure 1) trained on outputs from different analyzers (Soundgen vs Brown) and utterances of different lengths (short vs long). Notably, feature selection preceded training of our random forest classifier and involved the use of the Boruta algorithm on a reduced set of acoustic features (devoid of features with near zero variance, high missing data rates, or excessive intercorrelation) (Figure 2). All analyses were performed using R software, version 4 (R Foundation for Statistical Computing).^35^ Primary analyses used the Boruta,^31^ caret,^27^ ranger,^36^ epiR,^37^ and cutpointr^33^ packages for R. Statistical tests were 2-tailed, with α = .05 set as the significance threshold. Random forest classifiers used default hyperparameters from the ranger package (except for tree count [10 000 trees]).

## Results

### Descriptive Data

The final sample consisted of 65 neonates with a mean (SD) gestational age at birth of 36.6 (1.1) weeks; 36 infants (55.4%) were female, and 29 (44.6%) were male (Table). With regard to race, 1 infant (1.5%) was Asian, 3 (4.6%) were Black or African American, 50 (76.9%) were White, 6 (9.2%) were multiracial, and 5 (7.7%) were of unknown race; with regard to ethnicity, 15 (23.1%) were Hispanic, 49 (75.4%) were non-Hispanic, and 1 (1.5%) was of unknown ethnicity. We recorded 775 hours of audio across all 118 participants, resulting in 2.5 hours of usable cry recordings (mean [SD], 2.3 [2.3] min/neonate) for the final subset of 65 participants. The recording length did not differ between neonates with NOWS relative to neonates in the control group (mean [SD], 2.5 [2.1] min/neonate vs 2.3 [2.3] min/neonate; *t* 36.4  = 0.28; Cohen *d* = 0.08; *P* = .77).

**Table.** Maternal and Neonatal Demographic and Clinical Characteristics

Characteristic	Participants, No. (%)			*P* value
	Neonates with NOWS (n = 19)^a^	Neonates without NOWS (n = 46)^b^		
		Exposed to opioids (n = 7)	Not exposed to opioids (n = 39)	
**Maternal (prenatal)**				
Age, mean (SD), y	29.4 (4.8)	32.3 (3.3)	30.5 (5.7)	.34
Opioid substitute				
Methadone	12 (63.2)	3 (42.9)	0	<.001
Buprenorphine	7 (36.8)	3 (42.9)	0	
Other prescribed opioid	0	1 (14.3)	0	
None	0	0	39 (100)	
Illicit drug use^c^	6 (31.6)	0	0	<.001
Psychiatric medication use	6 (31.6)	2 (28.6)	0	.009
Less than high school education	6 (31.6)	2 (28.6)	11 (28.2)	.52
**Neonatal**				
Sex				
Female	8 (42.1)	4 (57.1)	24 (61.5)	.27
Male	11 (57.9)	3 (42.9)	15 (38.5)	
Gestational age, mean (SD), wk	40.1 (1.2)	39.7 (0.9)	39.4 (1.1)	.06
Birth weight, mean (SD), kg	3.1 (0.4)	3.4 (0.4)	3.4 (0.4)	.01
Birth length, mean (SD), cm	49.9 (2.4)	50.8 (1.9)	50.1 (5.2)	.73
Birth head circumference, mean (SD), cm	33.7 (1.3)	34.2 (1.2)	34.0 (3.6)	.53
Highest FNAST score, mean (SD)^d^	10 (2.3)	5 (1.2)	NA	NA

Abbreviations: FNAST, Finnegan Neonatal Abstinence Scoring Tool; NA, not available; NOWS, neonatal opioid withdrawal syndrome.

^a^ The NOWS group was composed of neonates exposed to opioids who developed NOWS.

^b^ The control group was composed of neonates exposed to opioids who did not develop NOWS and healthy infants who were not exposed to opioids.

^c^ Illicit drug use includes nonprescribed use of hallucinogens, cocaine, opioids, heroin, stimulants, cannabis, or sedatives.

^d^ Score range, 0-46, with higher scores indicating more severe withdrawal symptoms.

Mothers of neonates with NOWS vs neonates in the control group reported higher rates of illicit drug use (6 of 64 women [9.4%] vs 0 women; χ^2^ 1 = 12.18; *P* < .001) and higher use of psychiatric medication (6 of 65 women [9.2%] vs 2 of 65 women [3.1%]; χ^2^ 1 = 6.89; *P* = .009). Birth weight was also lower in neonates with NOWS relative to neonates in the control group (mean [SD], 3.1 [0.4] kg vs 3.4 [0.4] kg; *t* 32.49 = 2.73; Cohen *d* = 0.77; *P* = .01).

### Candidate Variable Selection

**Figure 3.**
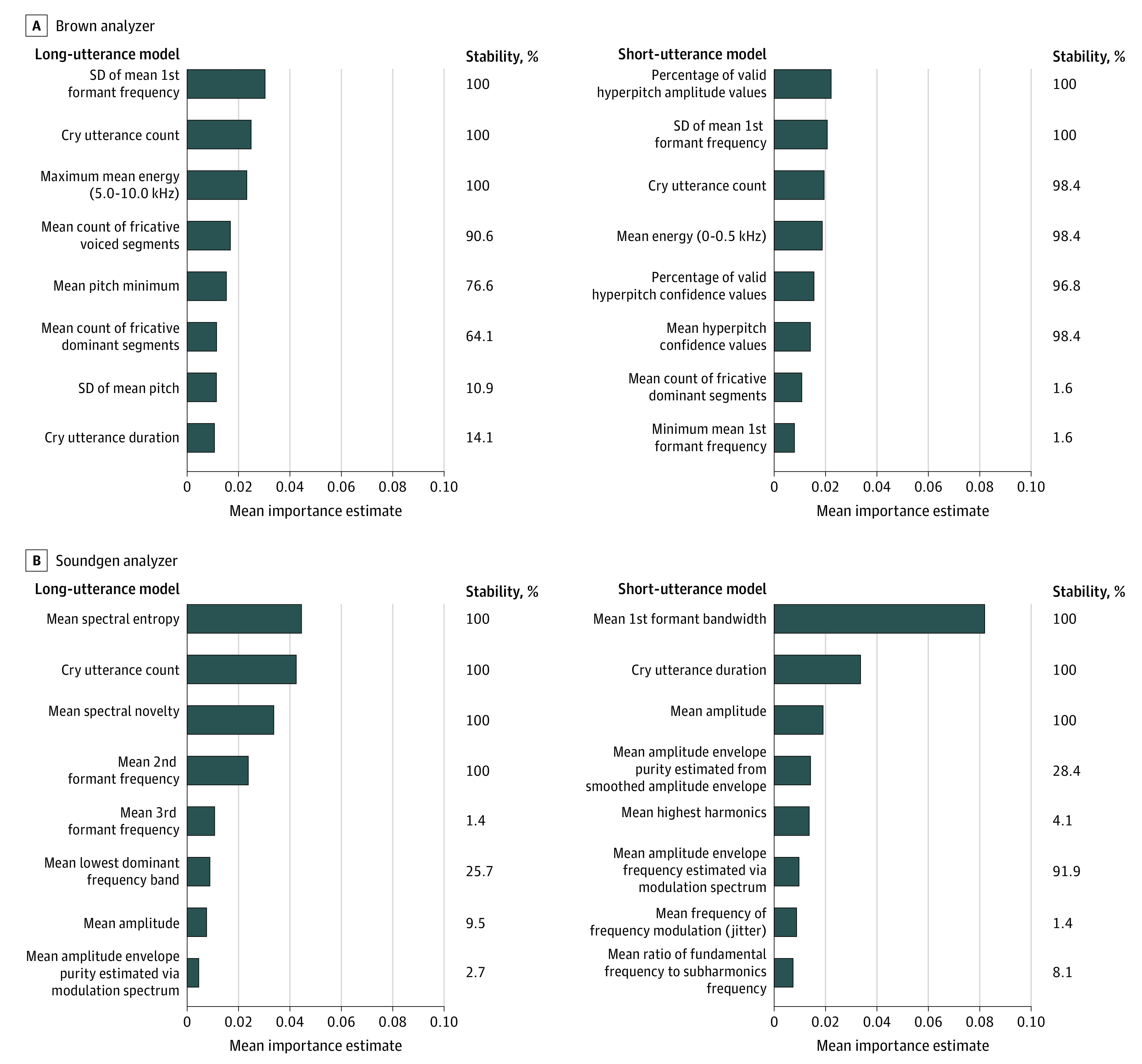
Acoustic Feature Importance and Stability Plots Mean importance estimates represent the relative contribution of a given variable to the final prediction across iterations of the cross-validation procedure. Variable stability was estimated by examining how frequently a given variable was retained for analysis during the feature selection step; for stability estimates, 100% indicates that a given variable was retained during all iterations of the cross-validation procedure. Both high importance and high stability are desirable.

The ensemble Boruta algorithms identified 16 variables from the Brown and Soundgen analyzers as well as 8 variables from the short-utterance model and 8 variables from the long-utterance model, for a total 32 variables (Figure 3). The important variables were those with both high stability and high importance. For the Brown analyzer, 10 of the 16 variables had greater than 90% stability and similar importance estimates and were considered important variables. For the Soundgen analyzer, 8 of the 16 variables had greater than 90% stability, 7 of which had similar importance estimates (with the exception of mean amplitude envelope frequency) and were considered important variables. Of the 10 important variables from the Brown analyzer, 3 were related to high pitch (hyperpitch), 2 were related to the first formant frequency, 2 were related to energy, 2 were related to the number of cry utterances, and 1 was related to fricatives. From the Soundgen analyzer, among 7 important variables, 2 were related to number of utterances, 1 was related to the first formant frequency, 1 was related to the second formant frequency, 1 was related to amplitude (energy), 1 was related to spectral entropy, and 1 was related to spectral novelty.

### Model Predictions

**Figure 4.**
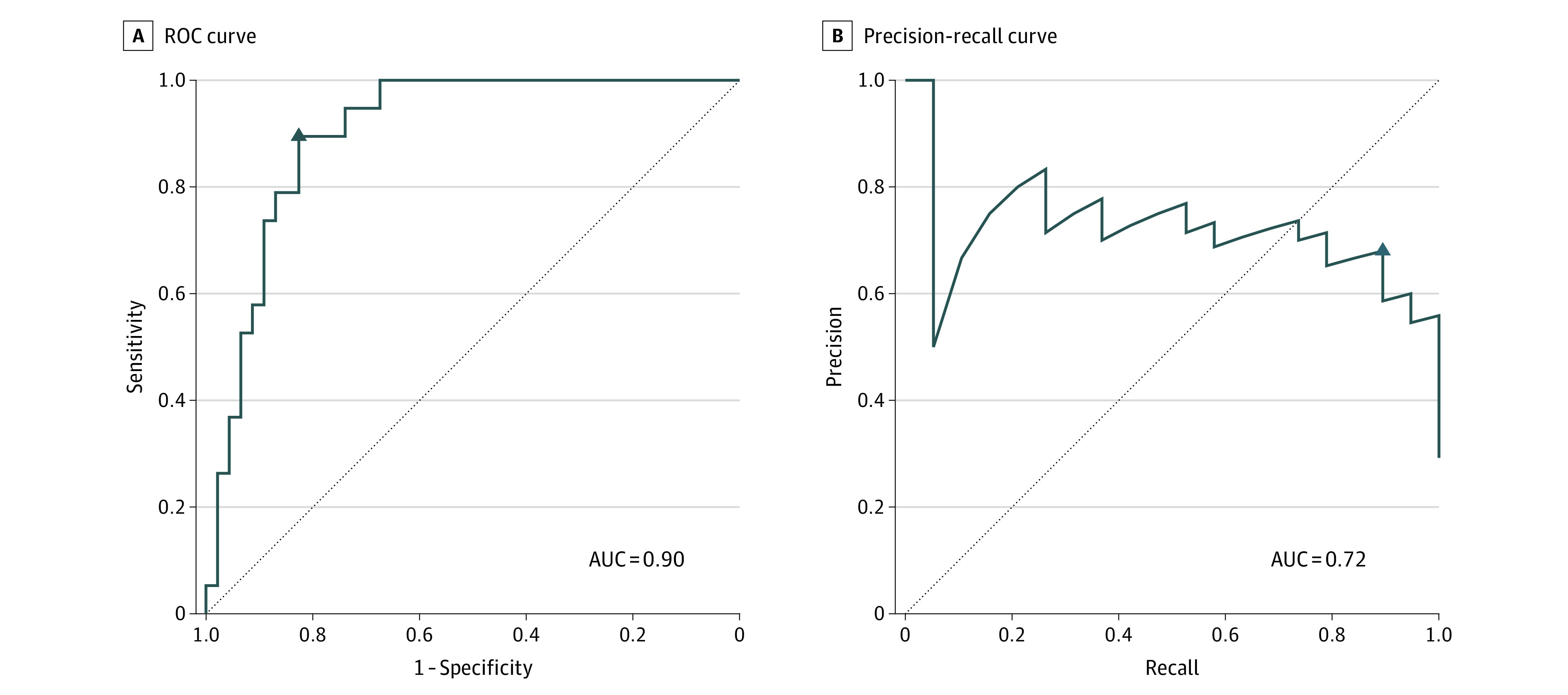
Receiver Operating Characteristic Curve and Precision-Recall Curve of the Final Prediction The triangle symbol indicates the point that was closest to perfect prediction (ie, values of 1 for sensitivity and 1 for specificity). In the precision-recall curve, precision is equivalent to positive predictive value, and recall is equivalent to sensitivity. AUC indicates area under the curve; ROC, receiver operating characteristic.

The mean of the predictions of stacked random forest classifiers predicted receipt of pharmacological treatment for NOWS with high diagnostic accuracy. The area under the curve (AUC) was 0.90 (95% CI, 0.83-0.98), accuracy was 0.85 (95% CI, 0.74-0.92), Cohen κ was 0.66 (95% CI, 0.47-0.85), sensitivity was 0.89 (95% CI, 0.67-0.99), specificity was 0.83 (95% CI, 0.69-0.92), positive predictive value (PPV) was 0.68 (95% CI, 0.46-0.85, and negative predictive value (NPV) was 0.95 (95% CI, 0.83-0.99) (Figure 4).

### Sensitivity Analyses

Predictions of the random forest classifiers remained accurate when excluding neonates with no prenatal illicit substance exposure (n = 39) from the ROC curve and confusion matrix (AUC, 0.85 [95% CI, 0.59-1.00]; accuracy, 0.88 [95% CI, 0.70-0.98]; Cohen κ, 0.72 [95% CI, 0.42-1.00]; sensitivity, 0.89 [95% CI, 0.67-0.99]; specificity, 0.86 [95% CI, 0.42-1.00]; PPV, 0.94 [95% CI, 0.73-1.00]; NPV, 0.75 [95% CI, 0.35-0.97]). Predictions were also accurate when excluding neonates with mothers receiving MAT who did not meet criteria for NOWS diagnosis (n = 7) from the ROC curve and confusion matrix (AUC, 0.91 [95% CI, 0.84-0.98]; accuracy, 0.84 [95% CI, 0.73-0.93]; Cohen κ, 0.67 [95% CI, 0.47-0.87]; sensitivity, 0.89 [95% CI, 0.67-0.99]; specificity, 0.82 [95% CI, 0.66-0.92]; PPV, 0.71 [95% CI, 0.49-0.87]; NPV, 0.94 [95% CI, 0.80-0.99]).

## Discussion

The assessment and management of NOWS has been problematic,^16,38^ in part because extant assessments rely on the subjective observations of health care professionals.^10,39^ The findings of this cohort study suggest that acoustic cry analysis has potential as an objective measure of opioid withdrawal in the neonate. Cross-validated random forest models trained using newborn cry acoustics were able to generate accurate predictions (AUC, 0.90; accuracy, 0.85; sensitivity, 0.89; specificity, 0.83). These results inform the feasibility of developing a NOWS algorithm based on cry acoustics that could improve the treatment and outcome of these infants.

Acoustic characteristics of the infant’s cry have been associated with prenatal opioid exposure^40,41^ and other medical conditions, including asphyxia, hyperbilirubinemia, trisomy anomalies, sudden infant death syndrome,^42^ prenatal drug exposure,^41^ autism,^43^ preterm birth,^44^ and other conditions,^45,46^ but these studies typically examined a limited subset of acoustic characteristics (most notably, pitch). Pitch is our perception of the fundamental frequency caused by vibration of the vocal folds and has received the most attention because it has intuitive appeal and because variation in pitch can be discriminated by listeners. However, as a single parameter associated with numerous conditions, it has limited diagnostic utility when used in isolation. Moreover, the reliability of listener ratings of pitch is low, and actual variation in fundamental frequency is high in relation to prenatal opioid exposure, even when measured through acoustic analysis.^40,41,47^ It is more likely that multidimensional aspects of cry acoustics have a role in the characterization of NOWS and other conditions, as suggested by a recent meta-analysis of cry characteristics and neurological dysfunction.^45^

Of the 17 important variables identified in our analysis, 7 were measures of frequencies in the vocal tract, including the fundamental frequency (pitch). Other frequencies included the first and second formants, which are frequency peaks or resonances as the cry sound travels upward through the vocal tract. Changes in the first formant frequency have been found in infants who died of sudden infant death syndrome,^48^ preterm infants,^49^ infants with hyperbilirubinemia,^50^ and infants with prenatal substance exposure.^41,51,52^ Changes in the second formant frequency have been reported in infants with prenatal substance exposure.^41,52^ Formant frequencies are determined by the shape and cross-sectional area of the vocal tract affected by cranial nerve activity.

Four of the important variables were the number of utterances (both short and long); these variables would generally correspond to the amount of crying, reflecting autonomic (including vagal) control. Cry utterance findings have been observed in preterm infants^48^ and infants with prenatal substance exposure.^41,53^ Two related measures of amplitude or energy, representing the extent of acoustic information in different ranges in the spectral domain and influencing loudness, are also under autonomic control. These measures have been associated with preterm birth^48,49^ and prenatal substance exposure.^41,48,51,53^ Fricatives are sounds caused by turbulent forced breath due to constriction of the vocal tract, which is affected by cranial nerve innervation of the vocal tract. Spectral entropy is a measure of the uniformity or quality of the signal. Spectral novelty is the identification of new or unknown data that a machine learning system is not aware of during training.^54^ Changes in measures of fricatives, spectral entropy, and spectral novelty have not been reported in infants at high risk of developing NOWS.

Our work was made possible by the combination of 2 major advances: state-of-the-art signal processing algorithms to quantify acoustic cry characteristics and machine learning methods to handle large data sets, including the array of acoustical information generated by the cry analyzers, which can model nonlinear associations via the underlying tree-based algorithm. In addition, the use of model stacking allowed us to use multiple analyzers and validate acoustic parameters across various platforms.

The field of NOWS is somewhat disorganized due to inconsistency regarding how NOWS is assessed and managed, lack of a standard treatment protocol, and a movement toward nonpharmacological interventions. However, common to these assessment tools (the FNAST, ESC, or consensus clinical definition) is that they include crying as a major feature, but crying is not measured objectively. Our findings suggest that the cries of infants with NOWS have distinct characteristics, constituting a cry signature that includes 17 variables, most of which cannot be detected by human perception. Acoustic cry analysis reflects the pathophysiological features of withdrawal, with implications for the brain stem, autonomic nervous system, and gut, and could serve as an objective biobehavioral marker of NOWS.

### Strengths and Limitations

This study has several strengths and limitations. The study involved the collection of more than 775 hours of audio recordings from neonates. Although we analyzed more than 2.5 hours of cry recordings, the number of neonates was small and confined to a single hospital. For this reason, we combined the 7 neonates exposed to opioids who did not meet criteria for treatment with healthy neonates who were not exposed to opioids into the same control group. Replication of these results in a larger multisite sample will be important. A larger sample would also allow for model probability calibration, thus producing an interpretable probability for the user (vs a binary prediction). The cries of the infants with NOWS that were collected with a recorder placed in the isolette resulted in substantial missing data and labor-intensive efforts to prepare cries for acoustic analysis. Cries of neonates exposed to opioids were recorded continuously, whereas cries of healthy neonates were recorded periodically; future work would benefit from fully standardizing the recording methods. Observed diagnostic estimates are favorable but could be improved (perhaps by incorporating additional infant information). Notably, future work would benefit from adjusting binary classification performance based on misclassification costs and/or expected clinical use (eg, screening for increased infant monitoring vs ruling out the need for pharmacological treatment).

It is also important to acknowledge that this approach was guided by analytic techniques rather than theory. We can only speculate about the mechanisms by which acoustic cry characteristics measure opioid withdrawal, and data-dependent approaches are sensitive to sample idiosyncrasies. We used extensive internal validation, and the fact that the Brown and Soundgen analyzers identified similar acoustic cry characteristics is a favorable finding, although external validation will be necessary to address whether results generalize beyond the present sample.

Our findings are based on evaluating whether cry acoustics corresponded to a measure (the FNAST) that has known limitations. However, the fact that cry acoustics and FNAST scores are associated suggests that the FNAST (and, by implication, other approaches such as the ESC or consensus report) has identified crying as an important dimension of neonatal opioid withdrawal but lacks objective measurement.

## Conclusions

The findings of this cohort study suggest that acoustic cry analysis using machine learning has potential as an objective biobehavioral measure of NOWS that could improve the assessment, diagnosis, and management of NOWS and facilitate standardized care for neonates with this condition.^55,56,57^

## Notes

Supplement. **eFigure.** Recruitment Flow Diagram
