# Supplementary material for: Newborn Cry Acoustics in the Assessment of Neonatal Opioid Withdrawal Syndrome Using Machine Learning
Source: JAMA Netw Open. 2022 Oct 27;5(10):e2238783. doi: 10.1001/jamanetworkopen.2022.38783 (PMC9614579; doi:10.1001/jamanetworkopen.2022.38783)
Supplement: Supplement. — eFigure. Recruitment Flow Diagram [file jamanetwopen-e2238783-s001.pdf]

Supplemental Online Content

Manigault AW, Sheinkopf SJ, Silverman HF, Lester BM. Newborn cry acoustics in the assessment of neonatal opioid withdrawal syndrome using machine learning. *JAMA Netw Open*. 2022;5(10):e2238783. doi:10.1001/jamanetworkopen.2022.38783

eFigure. Recruitment Flow Diagram

This supplemental material has been provided by the authors to give readers additional information about their work.

© 2022 Manigault AW et al. *JAMA Network Open*.

eFigure. Recruitment Flow Diagram

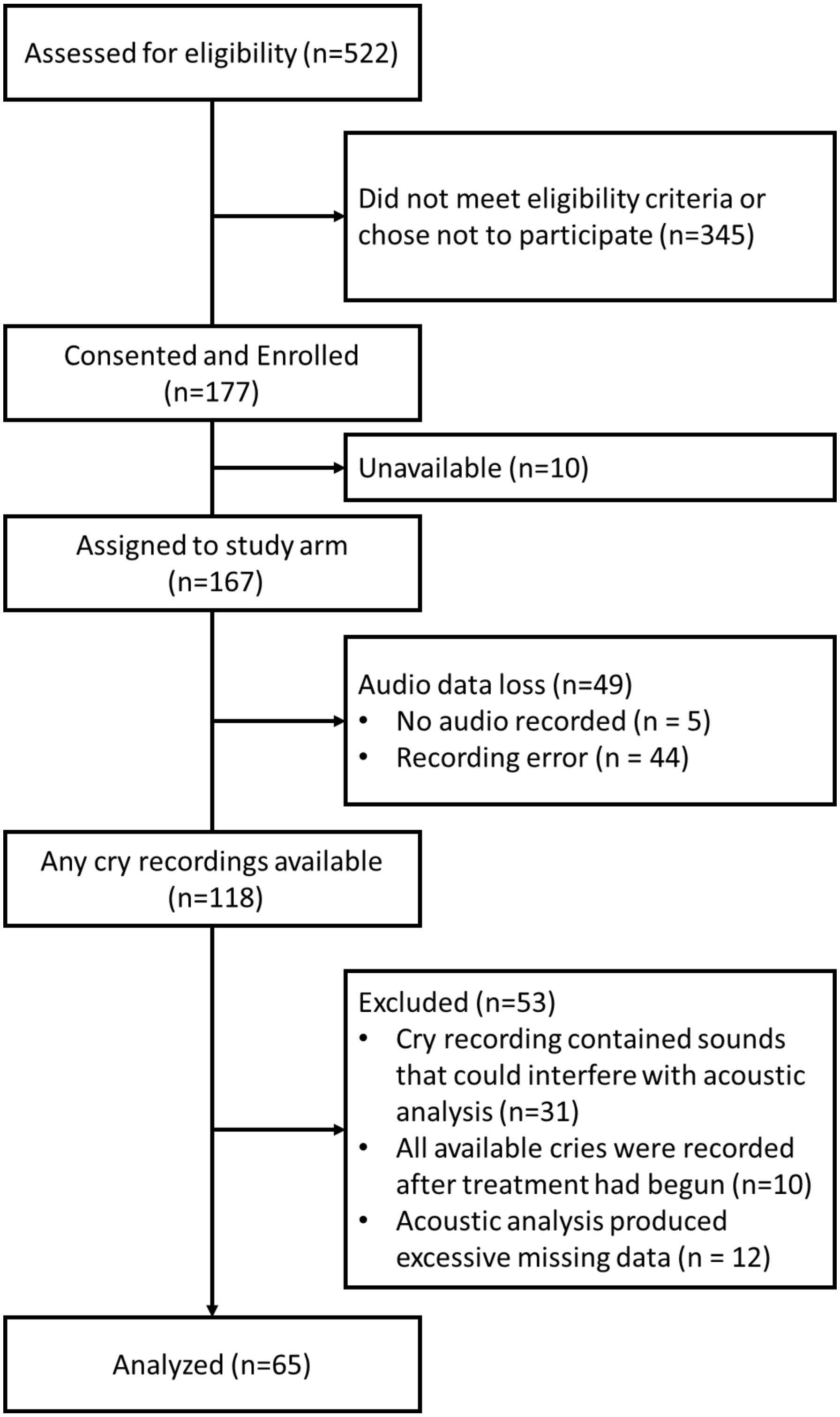

© 2022 Manigault AW et al. *JAMA Network Open*.
